# Stroke and Nursing Home care: a national survey of nursing homes

**DOI:** 10.1186/1471-2318-10-4

**Published:** 2010-01-27

**Authors:** Seamus Cowman, Maeve Royston, Anne Hickey, Frances Horgan, Hannah McGee, Desmond O'Neill

**Affiliations:** 1 Faculty of Nursing & Midwifery, Royal College of Surgeons in Ireland, St Stephen's Green, Dublin 2. Ireland; 2 Department of Psychology, Division of Population Health, Royal College of Surgeons in Ireland, St Stephen's Green, Dublin 2, Ireland; 3 School of Physiotherapy, Royal College of Surgeons in Ireland, St Stephen's Green, Dublin 2, Ireland; 4 Centre for Ageing Neurosciences and the Humanities, Trinity College, Dublin 2, Ireland

## Abstract

**Background:**

Although stroke is recognised as a major factor in admission to nursing home care, data is lacking on the extent and nature of the disabilities and dependency in nursing homes arising from stroke. A national study conducted in nursing homes can quantify the number of residents with stroke in nursing homes, their disability and levels of dependency.

**Methods:**

A cross-sectional survey research design was used. A total of 572 public and private nursing homes were identified nationally and a stratified random selection of 60 nursing homes with 3,239 residents was made. In half of the nursing homes (n = 30) efforts were made to interview all residents with stroke Survey instruments were used to collect data from residents with stroke and nursing home managers on demography, patient disability, and treatment.

**Results:**

Across all nursing homes (n = 60), 18% (n = 570) of the residents had previously had a stroke. In homes (n = 30), where interviews with residents with stroke (n = 257), only 7% (n = 18) residents were capable of answering for themselves and were interviewed. Data on the remaining 93% (n = 239) residents were provided by the nursing home manager. Nurse Managers reported that 73% of residents with stroke had a high level of dependency. One in two residents with stroke was prescribed antidepressants or sedative medication. Only 21% of stroke residents were prescribed anticoagulants, 42% antiplatelets, and 36% cholesterol lowering medications. Stroke rehabilitation guidelines were lacking and 68% reported that there was no formal review process in place.

**Conclusions:**

This study provides seminal findings on stroke and nursing home services in Ireland. We now know that one in six nursing home residents in a national survey are residents with a stroke, and have a wide range of disabilities. There is currently little or no structured care (beyond generic care) for stroke survivors who reside in nursing homes in Ireland.

## Background

Stroke constitutes a formidable burden of disability for patients, their carers and the community in general and is the third leading cause of death worldwide [1]. In the Republic of Ireland approximately 10,000 cases of acute stroke were admitted to hospital in 2005 [2], and it is estimated that over 30,000 people are living with a disability following a stroke.

While approximately half of survivors of an acute stroke make a complete recovery, a further 30% will make an incomplete recovery, although they will not necessarily require assistance with usual care activities. The remaining 20% will require assistance with at least one activity of living [3]. While hospital costs may account for up to 71% of total stroke care costs [4], the cost of long-term care is a major economic concern. A variety of long term-care arrangements are used by people who have lost physical or mental functioning as a result of stroke [5,6]. Options may include community-based care, institutional long-term nursing home care, self-care using assistive devices, or a combination of these. Costs are both direct (e.g. governments or individuals paying for nursing home care) and indirect (e.g. family members of the stroke patient quitting or reducing employment to provide home care).

It is now recognised that the way acute and rehabilitation stroke services are organised can have an important effect on patient outcome [7], and this has generated a major impetus for change in these services. Although continuity of services post discharge from acute care and/or rehabilitation following a stroke has commanded attention [8], there has not been an equivalent research focus on the needs of those with stroke in nursing homes. Many reports describe unmet service and information needs of stroke patients, their carers and families following discharge to the community from hospital [9]. Preliminary reports for those in nursing homes suggest major problems in terms of provision of rehabilitation and therapist input [10].

In recognition of a lack of reliable information across all sectors of care, including nursing homes the Irish Heart Foundation commissioned a first Irish National Audit of Stroke Care (INASC) [11]. The aim of the study was to conduct a national stroke audit of hospital and community stroke care, and to establish the current level and functioning of services available for the care of stroke patients in both the community and public acute hospitals, in the Republic of Ireland.

The community component of INASC involved a study of general practitioners (GPs); allied health professionals and public health nurses; patients and carers; and nursing homes. Each of the four elements of the study sought to document the views, experiences and needs of these key groups with regard to stroke management and care. The focus of this paper is to assess the prevalence of stroke in nursing homes in Ireland and the extent of disability and dependency of stroke patients.

### Nursing Home Facilities

In Ireland there are approximately 9,500 long-stay nursing home beds in the public sector and 18,000 in the private sector. The supply of long-term public care beds has remained relatively static in the last decade with a trend towards the development of private nursing home beds in a period of tax incentives and capital allowances. The number of nursing home beds in the private sector has increased from 10,500 in 1995 to 18,000 in 2007 [12]. In Ireland, those requiring long-term care as a result of a care episode in a general hospital are entitled to a fully-funded nursing home bed. Due to the lack of beds in public nursing homes, the Health Service Executive (HSE) often pays for public patients in private nursing homes to a varying extent. Most often medical services to nursing homes are provided through GPs, who are contracted for services. In private nursing homes it may be the case that some residents are attended to by their own GP.

### Nursing Home Residents

There is a dearth of research in Ireland on issues directly relating to care and services offered in nursing homes. Based on UK studies it is estimated that about 75% of residents in nursing homes are moderately or severely disabled [13]. A recent Irish census-based national study comparing older disabled people in the community and in nursing homes identified the presence of very high levels of disability among nursing home residents, with over 85% of residents having at least one recorded disability [14]. National Quality Standards for Residential Care Settings were published by the Health Information and Quality Authority in 2007 [15]. There is not yet a nationwide system of care needs assessment and provision in nursing homes, although a proposal exists for the introduction of a Minimum Data Set [15].

## Methods

A cross-sectional survey research design was used to investigate the experiences of nursing home managers and residents with stroke. A total of 572 public and private nursing homes were identified and a random selection was made of 60 nursing homes nationally, stratified by geographic location (20 urban and 40 rural). The selection resulted in 35 private nursing homes and 25 public nursing homes. Each nursing home proprietor or nominee was written to and their participation requested. The 60 nursing homes were visited and the manager, or nominee (all had nursing backgrounds) was interviewed by a member of the research team (MR). In half of the nursing homes (N = 30) selected randomly from the total sample of 60, interviews were requested with all residents with stroke, irrespective of co-morbidity.

### Measures

Two survey questionnaires (one for nursing home managers and one for residents) were developed and piloted with appropriate representative samples in advance of the main study. The content of the nursing home manager questionnaire comprised questions relating to nursing home profile; number of residents affected by stroke, number of residents who sustained a stroke since admission to the nursing home, access to treatment and services, and the specific challenges in providing optimal care for residents with stroke. For each resident with stroke, nursing home managers were questioned about level of dependency (including communication and swallowing difficulty and cognitive impairment) and risk of falls (See Table 1). It should be noted that no specific scales were used to measure risk of falls and results were based on the clinical judgment of the nursing home manager.

**Table 1:** Functional and cognitive problems experienced by post-stroke residents

Functional/Cognitive Problem identified	Public N = 398 %	Private N = 172 %	Overall (% of the total number) N = 570
**Communication Difficulty**	51	51	51
**Swallow difficulty**	54	45	52
**Cognitive impairment**	66	59	64
**Positioning needs**	87	80	85
**Limited independence**	85	89	86
**Risk of falls**	86	90	87
**Decreased independence in transfers (bed to chair and back)**	90	84	88
**Decreased balance**	90	85	86
**Poor mobility/Mobility needs**	93	87	83
**Residual weakness after stroke**	93	89	92

The resident questionnaire was administered by a member of the research team and addressed areas such as details of the stroke, functional capacity by means of the Barthel Index [16], psychological well-being by means of the Hospital Anxiety and Depression Scale (HADS) [17] and resident access to services (HeSSOP-2) [18]. Two questions from the Irish Census Household Survey (2002) were also included in the questionnaire incorporating questions relating to blindness, deafness, vision or hearing impairment and restriction to functional activities [14]. Of patients with stroke, deemed well enough by the nursing home manager to participate, all were interviewed by the researcher (MR). For those residents deemed too unwell or unable to be interviewed, a profile of functional capacity (Barthel Index) and levels of dependency was obtained with the co-operation of the nursing home manager. All data were collected from nursing home managers in a single interview. Ethical approval for the study was provided by the Research Ethics Committee of the Royal College of Surgeons in Ireland.

## Results

The sixty nursing homes surveyed had 3,239 residents. From the 60 nursing homes 18% (n = 570) of residents were residents with stroke. Less than 1% (n = 22) of nursing home residents had a stroke since admission to the nursing home. Of the 570 residents with stroke 83% (n = 471) were over 75 years and 15% (n = 87) were in the 65-74 age group. A small minority 2% (n = 12) were under 65 years. Overall one in six nursing home residents were residents with a stroke.

### Level of Dependency and Disability

In all of the nursing homes (n = 60) the nursing home manager was asked to identify levels of physical dependency of their residents with stroke, using a basic categorisation; mild (independent); moderate (needs some help) and severe (dependent). The resulting categorisation reflected the clinical judgment of the nursing home manager and was not based on a formal scale. Managers reported that 73% (n = 416) of residents with stroke had a high level of dependency. Approximately 22% (n = 125) were considered moderately dependent with only 5% (n = 29) being considered somewhat independent. Mobility needs were most common with over 86% (n = 490) of residents with a stroke having difficulties with balance and 87% (n = 496) deemed to be at risk of falls. An overview of the functional and cognitive problems experienced by stroke residents is outlined in Table 1.

In half of the nursing homes (n = 30), the aim was to interview all residents with stroke. In these randomly selected nursing homes there were a total of 257 residents with stroke. The nursing home manager deemed that only 18 residents with stroke were suitable for interview and these residents were interviewed by a member of the research team (MR), and completed the Barthel index and the HADS. For the remaining 239 residents with stroke, the nursing home manager was requested to complete the Barthel Index and the two questions from the Irish Census Household Inventory, on behalf of the residents.

Responses indicated that 39% (n = 94) of residents with stroke had one of the following conditions: blindness, deafness or a severe vision or hearing impairment. It was also identified that 81% (n = 193) of residents with stroke had difficulties in learning, memory and concentration and 94% (225) had difficulties in dressing, bathing or mobilising outside the home. None of the residents were considered to be capable of going outside of the nursing home alone to a shop or visit a doctor's surgery or to be able to work.

### Resident Self Report

In the 18 residents with stroke interviewed, levels of physical disability were reported as moderately severe to severe disability 89% (n = 16), moderate disability 6% (n = 1), and no significant disability 6% (n = 1). The scores obtained through the use of the HADS indicated that the majority of these residents with stroke were not depressed with 6 residents scoring as having possible or probable depression.

### Medications

Amongst residents with stroke (n = 570) across the 60 nursing homes, 53% (n = 301) were prescribed an antidepressant and 56% (n = 317) a sedative. Cardiovascular medication prescribing for residents with stroke included anticoagulants 21% (n = 119), anti-platelet medication 55% (n = 311) and antihypertensive medication 49% (n = 278). Cholesterol lowering medication was prescribed for 36% (n = 204) of residents.

### Communications and Monitoring of Care

Over one-third of nursing home managers 34% (n = 193) reported communication with acute hospitals as being poor, mainly because hospitals did not send a report and/or care plan when discharging the patient from the hospital to the nursing home. Prior to the transfer of a person with stroke to a nursing home, 55% (n = 312) of nursing homes required an additional assessment. No guidelines for stroke rehabilitation were reported by 92% (n = 522) and 68% (n = 386) identified that there was no formal review mechanism in place for patients with stroke.

## Discussion

Some stroke survivors require nursing home placement after their stroke episode and institutionalisation is considered to be one of the most adverse outcomes of stroke [19]. This first national survey goes some way to redressing the lack of available data on residents in nursing homes with stroke and stroke-associated disabilities. Based on the study sample, it is estimated conservatively that at least 4,000 Irish nursing home residents (1 in 6) are residents with a stroke. This is consistent with data from the US indicating that 20.4% of nursing home residents in 2009 have a diagnosis of stroke (http://www.cms.hhs.gov/MDSPubQIandResRep/, accessed 21 Oct 2009). Over 80% of stroke residents in nursing homes had a high level of dependency; this finding validates previous work based on census data that nursing homes are repositories of significant and complex disability [14]. There was a higher prevalence of residents with stroke in public nursing homes compared to private nursing homes. The precise reason for this is unclear, however it is noted that residents with stroke in public nursing homes generally had higher levels of functional and cognitive impairment than residents with stroke in private nursing homes.

The fact that 18% of nursing home residents had a diagnosis of stroke, an old age profile and high dependency levels represents a considerable service and care requirement. In a US population-based analysis, age and severity of stroke were independent predictors of nursing home residence after stroke [20]

### Disability and Rehabilitation

There is abundant research evidence that early post-stroke rehabilitation provides best results, including maintaining or improving functioning and quality of life after stroke. However, in this study standardised protocols for maintenance therapy and rehabilitation did not appear to be in place for patients transferred to nursing homes.

### Transitions/Long-term Care Issues

Differing healthcare policies and service organisation are associated with significant differences in processes of patient care [21]. The absence of guidelines for stroke rehabilitation and the lack of a formal review process for persons with stroke across the nursing home sector in this national survey are likely to have fostered inconsistency in approaches to stroke care and best practice. Care pathways incorporating multidisciplinary patterns of care are essential to successful rehabilitation in stroke care. In Ireland, a mechanism to support this was a proposed stroke liaison nurse scheme [22] with a planned 43 liaison nurses across all acute care hospitals by 2011. To date there have been few developments with the proposal.

This study illustrates a lack of strategy, fragmentation and an 'ad hoc' approach to service delivery in stroke care. A whole systems approach is essential to planning, delivery and ensuring capacity in the services, so as to offer people affected by stroke, choice, quality services and access to care. One key component for achieving this is to have clear and uniform resident assessment outcomes for quality of care standards in nursing homes. One mechanism to achieve this is a minimum data set. The Minimum Data Set (MDS) has been developed as part of a Resident Assessment Instrument to assist US nursing homes in developing a comprehensive care plan for each resident [23]. It is now used in the US to provide regular assessment of every resident in State-licensed facilities. In Ireland, following a public enquiry into poor standards of nursing home care [24], a recommendation was made for the introduction of the MDS in all nursing homes.

### Living on the Margins of Care

From a therapeutic perspective many residents with stroke are on the margins of services and care, in particular given that older people often have concomitant chronic diseases which may require medication. A UK primary care study [25] identified 'that poor monitoring of disease and unnecessary drug prescribing are more likely to occur in nursing home residents than in people living at home, even after controlling for co-morbidity and prescribed medication'. A review of medication prescribing for residents with stroke formed a small part of this overall study and it is difficult to draw any definitive conclusions. What is noted however is that the patterns for cardiovascular medication prescribing in nursing homes for residents with stroke are much lower than a hospital discharge group (N = 2,173) of consecutive cases of stroke across Irish hospitals, which reported discharge prescribing rates for stroke as 78% anti-hypertensives, 85% anti-platelets/anti-thrombotics and 70% lipid lowering agents [11]. In nursing homes, medication prescribing practices appeared to be below best standards and frequency of medical reviews was not quantified. The finding that over 50% of post-stroke residents in our study were on antidepressant medication should be noted. However, it should be observed that only a small proportion of residents with stroke were capable of responding to the HADS inventory.

There are a number of limitations to the study. The most important limitation of the study relates to the scope of the study. As part of a first national audit, primacy was given to collecting a small amount of information across a relatively large set of constructs. Thus there was a lack of depth to many areas of investigation, for example, there was a shortage of supplementary information to understand how low levels of depression, as measured by the HADS, could be reconciled with high levels of use of antidepressants. While it would have been valuable to interview more residents with stroke in detail, every effort was made, consistent with the ethical protocol, to include all patients capable of participating. The research team was conscious of potential bias in collecting data from nursing home management. One experienced nurse researcher undertook all interviews in accordance with the study protocol.

## Conclusions

This study has identified a number of challenges to providing optimal health and social care to residents who have experienced stroke and are in long-term care. Gaps in services and limited resources were reported for this vulnerable population group as well as lack of a uniform system for recording disability and clinical/functional status. Access to appropriate health professionals following a standardised needs assessment is lacking and must be redressed. Standardised care plans and ongoing education for relevant health professionals is also required to ensure stroke-relevant support and therapy for those with stroke who enter nursing homes.

There needs to be an increased societal awareness that stroke patients who live in nursing homes are community residents whose home address happens to be a nursing home. They thus should feature as community residents in any planning or service developments. The findings of this study have little international data against which to benchmark quality of care. Nursing home residents have been relatively invisible in stroke care considerations to date. Use of the instruments developed here in other settings would provide information for comparison of best practice across countries and regions and enable fruitful discussion of ways to improve care for nursing home residents who have experienced a stroke.

In summary, one in six nursing home residents in a national study has had a stroke. As a patient group they present with high levels of functional and cognitive impairment. Their care needs are not currently addressed in a systematic manner. Service improvements must come from a combination of enhanced multidisciplinary team efforts and related resources across the entire community and nursing home sector drawing on the expertise and insights of stroke physicians, rehabilitationists, nurses and allied health professionals. National audits of stroke should extend their current focus on hospital care to a wider view of all those affected by stroke, including those who become nursing home residents as a result of their stroke [26].

## Competing interests

The authors declare that they have no competing interests.

## Authors' contributions

All authors contributed to all elements in the preparation of this paper including a review of three drafts of the paper. All authors have read and approved the final manuscript

## Pre-publication history

The pre-publication history for this paper can be accessed here:

http://www.biomedcentral.com/1471-2318/10/4/prepub
